# Supplementary material for: The risk of Long Covid symptoms: a systematic review and meta-analysis of controlled studies
Source: Nat Commun. 2025 May 7;16:4249. doi: 10.1038/s41467-025-59012-w (PMC12059169; doi:10.1038/s41467-025-59012-w)
Supplement: Supplementary file 1 — Supplementary Information [file 41467_2025_59012_MOESM1_ESM.pdf]

Supplementary Table 1. PRISMA Checklist

Section and Topic	Item #	Checklist item	Location where item is reported
**TITLE**			
Title	1	Identify the report as a systematic review.	Page 1
**ABSTRACT**			
Abstract	2	See the PRISMA 2020 for Abstracts checklist.	Page 4
**INTRODUCTION**			
Rationale	3	Describe the rationale for the review in the context of existing knowledge.	Pages 5-6
Objectives	4	Provide an explicit statement of the objective(s) or question(s) the review addresses.	Page 6
**METHODS**			
Eligibility criteria	5	Specify the inclusion and exclusion criteria for the review and how studies were grouped for the syntheses.	Page 15-16
Information sources	6	Specify all databases, registers, websites, organisations, reference lists and other sources searched or consulted to identify studies. Specify the date when each source was last searched or consulted.	Page 17
Search strategy	7	Present the full search strategies for all databases, registers and websites, including any filters and limits used.	Supplementary file, Table S2
Selection process	8	Specify the methods used to decide whether a study met the inclusion criteria of the review, including how many reviewers screened each record and each report retrieved, whether they worked independently, and if applicable, details of automation tools used in the process.	Page 17-18
Data collection process	9	Specify the methods used to collect data from reports, including how many reviewers collected data from each report, whether they worked independently, any processes for obtaining or confirming data from study investigators, and if applicable, details of automation tools used in the process.	Page 18
Data items	10a	List and define all outcomes for which data were sought. Specify whether all results that were compatible with each outcome domain in each study were sought (e.g. for all measures, time points, analyses), and if not, the methods used to decide which results to collect.	Page 19
	10b	List and define all other variables for which data were sought (e.g. participant and intervention characteristics, funding sources). Describe any assumptions made about any missing or unclear information.	Page 19
Study risk of bias assessment	11	Specify the methods used to assess risk of bias in the included studies, including details of the tool(s) used, how many reviewers assessed each study and whether they worked independently, and if applicable, details of automation tools used in the process.	Page 18
Effect measures	12	Specify for each outcome the effect measure(s) (e.g. risk ratio, mean difference) used in the synthesis or presentation of results.	Page 19
Synthesis methods	13a	Describe the processes used to decide which studies were eligible for each synthesis (e.g. tabulating the study intervention characteristics and comparing against the planned groups for each synthesis (item #5)).	Page 19
	13b	Describe any methods required to prepare the data for presentation or synthesis, such as handling of missing summary	Page 19

Section and Topic	Item #	Checklist item	Location where item is reported
		statistics, or data conversions.	
	13c	Describe any methods used to tabulate or visually display results of individual studies and syntheses.	Page 19
	13d	Describe any methods used to synthesize results and provide a rationale for the choice(s). If meta-analysis was performed, describe the model(s), method(s) to identify the presence and extent of statistical heterogeneity, and software package(s) used.	Page 19
	13e	Describe any methods used to explore possible causes of heterogeneity among study results (e.g. subgroup analysis, meta-regression).	Page 19
	13f	Describe any sensitivity analyses conducted to assess robustness of the synthesized results.	N/A
Reporting bias assessment	14	Describe any methods used to assess risk of bias due to missing results in a synthesis (arising from reporting biases).	N/A
Certainty assessment	15	Describe any methods used to assess certainty (or confidence) in the body of evidence for an outcome.	N/A
**RESULTS**			
Study selection	16a	Describe the results of the search and selection process, from the number of records identified in the search to the number of studies included in the review, ideally using a flow diagram.	Figure 1.
	16b	Cite studies that might appear to meet the inclusion criteria, but which were excluded, and explain why they were excluded.	N/A
Study characteristics	17	Cite each included study and present its characteristics.	Supplementary file, Table S3
Risk of bias in studies	18	Present assessments of risk of bias for each included study.	Supplementary file, Table S4
Results of individual studies	19	For all outcomes, present, for each study: (a) summary statistics for each group (where appropriate) and (b) an effect estimate and its precision (e.g. confidence/credible interval), ideally using structured tables or plots.	Figure 2 Supplementary figures 1-3.
Results of syntheses	20a	For each synthesis, briefly summarise the characteristics and risk of bias among contributing studies.	Supplementary file, Table S4.
	20b	Present results of all statistical syntheses conducted. If meta-analysis was done, present for each the summary estimate and its precision (e.g. confidence/credible interval) and measures of statistical heterogeneity. If comparing groups, describe the direction of the effect.	Figure 2 Supplementary figures 1-3.
	20c	Present results of all investigations of possible causes of heterogeneity among study results.	Figure 2 Supplementary figures 1-3.
	20d	Present results of all sensitivity analyses conducted to assess the robustness of the synthesized results.	N/A
Reporting biases	21	Present assessments of risk of bias due to missing results (arising from reporting biases) for each synthesis assessed.	N/A
Certainty of	22	Present assessments of certainty (or confidence) in the body of evidence for each outcome assessed.	N/A

Section and Topic	Item #	Checklist item	Location where item is reported
evidence			
**DISCUSSION**			
Discussion	23a	Provide a general interpretation of the results in the context of other evidence.	Pages 10 - 15
	23b	Discuss any limitations of the evidence included in the review.	Pages 10 – 15
	23c	Discuss any limitations of the review processes used.	Pages 10 - 15
	23d	Discuss implications of the results for practice, policy, and future research.	Pages 10 - 15
**OTHER INFORMATION**			
Registration and protocol	24a	Provide registration information for the review, including register name and registration number, or state that the review was not registered.	Page 15
	24b	Indicate where the review protocol can be accessed, or state that a protocol was not prepared.	Page 15
	24c	Describe and explain any amendments to information provided at registration or in the protocol.	N/A
Support	25	Describe sources of financial or non-financial support for the review, and the role of the funders or sponsors in the review.	Page 25
Competing interests	26	Declare any competing interests of review authors.	Page 25-26
Availability of data, code and other materials	27	Report which of the following are publicly available and where they can be found: template data collection forms; data extracted from included studies; data used for all analyses; analytic code; any other materials used in the review.	Page 19

From: Page MJ, McKenzie JE, Bossuyt PM, Boutron I, Hoffmann TC, Mulrow CD, et al. The PRISMA 2020 statement: an updated guideline for reporting systematic reviews. BMJ 2021;372:n71. doi: 10.1136/bmj.n71

For more information, visit: http://www.prisma-statement.org/

Supplementary Table 2. Search Term Table

String	Search Terms
COVID terms	covid* or ncov* or novel coronavirus or novel betacoronavirus or sars-ncov-2 or sars-cov-2
Any of these within three words of any of the COVID terms	Long, chronic, long term, long-term, longterm, sequela*, post acute, post-acute, postacute, long haul*, long-haul*, longhaul*, survivor*, on going, on-going, ongoing
Any of these within five words of any of the COVID terms	persist*, recover*, discharg*, follow*, prolong*
Any of these combinations	post-covid syndrome, postcovid syndrome, post covid syndrome<br><br>symptomatic within three words of any of the COVID terms, but also with chronic, and any of long term, long-term, longterm<br><br>Any of chronic*, long term, long-term, longterm within three words of any of post covid, post-covid, postcovid

Supplementary Table 3. Study and patient characteristics.

Author	Total # of participants (Control)	Total # of participants (COVID)	Sex (% males) Control	Sex (% males) COVID	Age (years) Control	Age (years) COVID	Hospital Status	Time to follow up (mean/ median days)	Continent
Adler et al., 2022[1]	1936	819	43	40	48	47	Mixed	155	Asia
Ahn et al., 2023[2]	105	106	61	63	2	3	Non-hospitalised	182	Asia
Albtoosh et al., 2022[3]	347	125	64	50	41	39	Mixed	360	Africa
Ali et al., 2022[4]	439	439	48	51	38	38	Mixed	180	Asia
Askarian et al., 2024[5]	449	1561	30	37	42	41	Mixed	28	Asia
Barboza-Solis et al., 2024[6]	934	193	NR	NR	>18	>18	Mixed	>273	North America
Bergia et al., 2022[7]	98	451	57	55	<18	<18	Mixed	>84	Europe
Binswanger et al., 2024[8]	692	1537	NR	NR	>18	>18	Mixed	28	North America
Blomberg, et al. 2021[9]	43	293	35	49	42	47	Mixed	183	Europe
Boscolo-Rizzo et al., 2021[10]	100	100	61	61	49	49	Non-hospitalised	401	Europe
Cai et al., 2023[11]	979	450	63	57	40	42	Hospitalised	183	Asia
DeBruijn et al., 2024[12]	2726	10993	*	*	>18	>18	Mixed	>90	Europe
Desgranges et al., 2022[13]	89	418	37	38	36	41	Non-hospitalised	150	Europe
Fjelltveit et al., 2023[14]	189	233	34	47	41	44	Non-hospitalised	365	Europe
Funk et al., 2022[15]	380	391	52	54	<18	<18	Hospitalised	90	Multiple
	1321	1295	47	52	<18	<18	Non-hospitalised	90	Multiple
Golla et al., 2023[16]	280	320	61	51	38	38	Hospitalised	183	Asia
Hastie et al., 2023[17]	3974	6538	NR	NR	>16	>16	Mixed	538	Europe
Hernandez-Romieu et al., 2022 [18]	320902	18419	41	46	>20	>20	Hospitalised	31-150	North America
	1061692	128251	38	37	>20	>20	Non-hospitalised	31-150	North America
	47439	1217	50	49	<20	<20	Hospitalised	31-150	North America
	250073	24586	51	48	<20	<20	Non-hospitalised	31-150	North America
Horberg et al., 2022[19]	70293	28118	43	43	NR	NR	Mixed	>30	North America
Hosozawa et al., 2024[20]	1341	1800	53	54	5-17	5-17	Non-hospitalised	60	Asia
Huang et al., 2022[21]	1127	1127	54	54	59	59	Hospitalised	685	Asia
Huynh et al., 2023[22]	55	150	NR	NR	<16	<16	Hospitalised	28	Asia
Iba et al., 2024[23]	6318	8392	40	42.1	42	42	Mixed	168	Asia
Khullar et al., 2023[24]	71222	13106	NR	NR	>20	>20	Hospitalised	31-180	North America
Li et al., 2023[25]	471	169	53	54	6	6	Mixed	>90	Asia

Marasco et al., 2022[26]	258	530	63	59	52	51	Hospitalised	28	Europe
Nehme et al., 2022[27]	1160	287	40	35	46	44	Mixed	367	Europe
Newlands et al., 2023[28]	440	561	39	36	18-20	18-20	Non-hospitalised	213	Europe
Noviello et al., 2022[29]	183	164	39	60	40	44	Mixed	146	Europe
Nugawela et al., 2022[30]	3893	3246	37	37	11-17	11-17	Non-hospitalised	91	Europe
Pagen et al., 2023[31]	345	2322	NR	NR	18-81	18-81	Mixed	>90	Europe
Park, et al. 2021[32]	292674	6934	45	39	NR	NR	Mixed	183	Asia
Pihlaja et al., 2023[33]	50	155	51	44	55	53	Mixed	183	Europe
PintoPereira et al., 2023[34]	7474	8060	38	38	11-17	11-17	Non-hospitalised	365	Europe
Radtke, et al. 2021[35]	1246	109	46	47	12	11	Non-hospitalised	>84	Europe
Rao et al., 2022[36]	599393	59893	53	51	8	9	Mixed	140	North America
Rivera-Izquierdo et al., 2022[37]	453	453	47	57	60	61	Hospitalised	365	Europe
Roge, et al. 2021[38]	142	236	54	56	2	10	Mixed	74	Europe
Seery et al., 2023[39]	577	639	52	53	8	7	Hospitalised	>90	South America
Shah et al., 2023[40]	18098	11015	37	38	54	51	Mixed	>84	Europe
Sneller et al., 2022[41]	120	189	45	45	51	50	Mixed	149	North America
Subramanian et al., 2022[42]	1501689	384137	45	45	44	44	Non-hospitalised	106	Europe
Thors et al., 2024[43]	602	643	52	55	10	10	Non-hospitalised	283	Europe
Tisler et al., 2022[44]	15511	3949	46	46	65	65	Hospitalised	295	Europe
VanderMaaden et al., 2023[45]	2445	6614	32	37	54	52	Mixed	91	Europe
Van Herck et al., 2024[46]	2028	6381	51	41	58	51	Mixed	304	Europe
Xiong et al., 2021[47]	184	538	52	46	50	52	Hospitalised	97	Asia
Xu et al., 2022[48]	5606761	16764	90	94	63	68	Hospitalised	408	North America
	5606761	131915	90	90	63	63	Non-hospitalised	408	North America
Zhang et al., 2024[49]	2020829	262400	NR	NR	>20	>20	Non-hospitalised	≥30	North America
	662295	44926	NR	NR	>20	>20	Hospitalised	≥30	North America
	511383	58196	NR	NR	≤19	≤19	Non-hospitalised	≥30	North America
	91933	2725	NR	NR	≤19	≤19	Hospitalised	≥30	North America
Zhao et al., 2024[50]	113	257	54	30	48	49	Mixed	340	Europe

* Omicron and delta variant data was combined for the meta-analysis; age was reported as median thus it was not possible to combine ages.

Supplementary Table 4. Risk of Bias Summary for Included Studies (Quality Assessment)

	Selection				Comparability	Outcome			Study Score	Overall Study Rating
	Representativeness of the Exposed Cohort	Selection of the Non-Exposed Cohort	Ascertainment of Exposure	Demonstration that outcome of interest was not present at start of study	Comparability of cohorts*	Assessment of outcome	Follow-up long enough for outcomes to occur?	Adequacy of follow Up of cohorts		
Adler et al., 2022	Somewhat representative of the average patient/community member with Long Covid (eg, age, social class, ethnicity etc.) in the community	Drawn from the same community as the exposed cohort	Secure record (eg medical records)	No	Both of the above	Self-report (i.e., no reference to original medical records or laboratory reports to confirm the outcome)	Yes (i.e., >4 weeks)	No statement	7	Low Risk
Ahn et al., 2023	Somewhat representative of the average patient/community member with Long Covid (eg, age, social class, ethnicity etc.) in the community	Drawn from the same community as the exposed cohort	Secure record (eg medical records)	No		Record linkage (e.g., identified through ICD codes on database records)	Yes (i.e., >4 weeks)	No statement	5	Medium Risk
Albtoosh et al., 2022	Somewhat representative of the average patient/community member with Long Covid (eg, age, social class, ethnicity etc.) in the community	Drawn from the same community as the exposed cohort	Secure record (eg medical records)	No	Both of the above	Self-report (i.e., no reference to original medical records or laboratory reports to confirm the outcome)	Yes (i.e., >4 weeks)	No statement	7	Low Risk
Ali et al., 2022	Somewhat representative of the average patient/community member with Long Covid (eg, age,	Drawn from the same community as the exposed cohort	Secure record (eg medical records)	No		Self-report (i.e., no reference to original medical records or laboratory reports	Yes (i.e., >4 weeks)	No statement	4	Medium Risk

	social class, ethnicity etc.) in the community					to confirm the outcome)				
Askarian et al., 2024	Somewhat representative of the average patient/community member with Long Covid (eg, age, social class, ethnicity etc.) in the community	Drawn from the same community as the exposed cohort	Written self-report	No	Both of the above	Self-report (i.e., no reference to original medical records or laboratory reports to confirm the outcome)	Yes (i.e., >4 weeks)	No statement	6	Medium Risk
Barboza-Solis et al., 2024	Somewhat representative of the average patient/community member with Long Covid (eg, age, social class, ethnicity etc.) in the community	Drawn from the same community as the exposed cohort	Secure record (eg medical records)	No	Both of the above	Self-report (i.e., no reference to original medical records or laboratory reports to confirm the outcome)	Yes (i.e., >4 weeks)	Follow up rate < 80% and no description of those lost	7	Low Risk
Bergia et al., 2022	Somewhat representative of the average patient/community member with Long Covid (eg, age, social class, ethnicity etc.) in the community	Drawn from the same community as the exposed cohort	Secure record (eg medical records)	No		Self-report (i.e., no reference to original medical records or laboratory reports to confirm the outcome)	Yes (i.e., >4 weeks)	No statement	5	Medium Risk
Binswanger et al., 2024	Somewhat representative of the average patient/community member with Long Covid (eg, age, social class, ethnicity etc.) in the community	Drawn from the same community as the exposed cohort	Secure record (eg medical records)	No	Both of the above	Self-report (i.e., no reference to original medical records or laboratory reports to confirm the outcome)	Yes (i.e., >4 weeks)	Subjects lost to follow up unlikely to introduce bias (i.e., follow up rate > 80%), or	8	Low Risk

								description provided of those lost		
Blomberg, et al., 2021	Somewhat representative of the average patient/community member with Long Covid (eg, age, social class, ethnicity etc.) in the community	Drawn from the same community as the exposed cohort	Secure record (eg medical records)	No	Both of the above	Self-report (i.e., no reference to original medical records or laboratory reports to confirm the outcome)	Yes (i.e., >4 weeks)	Subjects lost to follow up unlikely to introduce bias (i.e., follow up rate > 80%), or description provided of those lost	7	Low Risk
Boscolo-Rizzo et al., 2021	Somewhat representative of the average patient/community member with Long Covid (eg, age, social class, ethnicity etc.) in the community	Drawn from the same community as the exposed cohort	Secure record (eg medical records)	No	Both of the above	Record linkage (e.g., identified through ICD codes on database records)	Yes (i.e., >4 weeks)	No statement	7	Low Risk
Cai et al., 2023	Somewhat representative of the average patient/community member with Long Covid (eg, age, social class, ethnicity etc.) in the community	Drawn from the same community as the exposed cohort	Secure record (eg medical records)	No	Both of the above	Self-report (i.e., no reference to original medical records or laboratory reports to confirm the outcome)	Yes (i.e., >4 weeks)	No statement	7	Low Risk

deBruijn et al., 2024	Somewhat representative of the average patient/community member with Long Covid (eg, age, social class, ethnicity etc.) in the community	Drawn from the same community as the exposed cohort	Secure record (eg medical records)	No	Both of the above	Self-report (i.e., no reference to original medical records or laboratory reports to confirm the outcome)	Yes (i.e., >4 weeks)	No statem ent	7	Low Risk
Desgran ges et al., 2022	Somewhat representative of the average patient/community member with Long Covid (eg, age, social class, ethnicity etc.) in the community	Drawn from the same community as the exposed cohort	Secure record (eg medical records)	No	Both of the above	Self-report (i.e., no reference to original medical records or laboratory reports to confirm the outcome)	Yes (i.e., >4 weeks)	No statem ent	6	Medium Risk
Fjelltveit et al., 2023	Somewhat representative of the average patient/community member with Long Covid (eg, age, social class, ethnicity etc.) in the community	Drawn from the same community as the exposed cohort	Secure record (eg medical records)	No	Study controls for age and sex in the analysis	Record linkage (e.g., identified through ICD codes on database records)	Yes (i.e., >4 weeks)	Subject s lost to follow up unlikely to introdu ce bias (i.e., follow up rate > 80%), or descrip tion provide d of those lost	7	Low Risk
Funk et al., 2022	Somewhat representative of the average patient/community member with Long Covid (eg, age,	Drawn from the same community as the exposed cohort	Secure record (eg medical records)	No	Study controls for age and sex in the analysis	Record linkage (e.g., identified through ICD codes on database records)	Yes (i.e., >4 weeks)	Subject s lost to follow up unlikely	7	Low Risk

	social class, ethnicity etc.) in the community							to introduce bias (i.e., follow up rate > 80%), or description provided of those lost		
Golla et al., 2023	Somewhat representative of the average patient/community member with Long Covid (eg, age, social class, ethnicity etc.) in the community	Drawn from the same community as the exposed cohort	Secure record (eg medical records)	Yes		Self-report (i.e., no reference to original medical records or laboratory reports to confirm the outcome)	Yes (i.e., >4 weeks)	Subjects lost to follow up unlikely to introduce bias (i.e., follow up rate > 80%), or description provided of those lost	6	Medium Risk
Hastie et al., 2023	Truly representative of the average patient/community member with Long Covid (eg, age, social class, ethnicity etc.) in the community	Drawn from the same community as the exposed cohort	Secure record (eg medical records)	No	Both of the above	Self-report (i.e., no reference to original medical records or laboratory reports to confirm the outcome)	Yes (i.e., >4 weeks)	No statement	7	Low Risk

Hernandez-Romieu et al., 2022	Somewhat representative of the average patient/community member with Long Covid (eg, age, social class, ethnicity etc.) in the community	Drawn from the same community as the exposed cohort	Secure record (eg medical records)	Yes	Both of the above	Record linkage (e.g., identified through ICD codes on database records)	Yes (i.e., >4 weeks)	No statement	8	Low Risk
Horberg et al., 2022	Somewhat representative of the average patient/community member with Long Covid (eg, age, social class, ethnicity etc.) in the community	Drawn from the same community as the exposed cohort	Secure record (eg medical records)	Yes	Both of the above	Record linkage (e.g., identified through ICD codes on database records)	Yes (i.e., >4 weeks)	No statement	8	Low Risk
Hosozawa et al., 2024	Somewhat representative of the average patient/community member with Long Covid (eg, age, social class, ethnicity etc.) in the community	Drawn from the same community as the exposed cohort	Secure record (eg medical records)	No	Both of the above	Self-report (i.e., no reference to original medical records or laboratory reports to confirm the outcome)	Yes (i.e., >4 weeks)	Subjects lost to follow up unlikely to introduce bias (i.e., follow up rate > 80%), or description provided of those lost	8	Low Risk
Huang et al., 2022	Somewhat representative of the average patient/community member with Long Covid (eg, age,	Drawn from the same community as the exposed cohort	Secure record (eg medical records)	Yes	Both of the above	Self-report (i.e., no reference to original medical records or laboratory reports	Yes (i.e., >4 weeks)	Follow up rate < 80% and no description of	7	Low Risk

	social class, ethnicity etc.) in the community					to confirm the outcome)		those lost		
Huynh et al., 2023	Somewhat representative of the average patient/community member with Long Covid (eg, age, social class, ethnicity etc.) in the community	Drawn from the same community as the exposed cohort	Secure record (eg medical records)	No		No description	Yes (i.e., >4 weeks)	No statement	4	Medium Risk
Iba et al., 2024	Somewhat representative of the average patient/community member with Long Covid (eg, age, social class, ethnicity etc.) in the community	Drawn from the same community as the exposed cohort	Secure record (eg medical records)	No	Both of the above	Self-report (i.e., no reference to original medical records or laboratory reports to confirm the outcome)	Yes (i.e., >4 weeks)	Subjects lost to follow up unlikely to introduce bias (i.e., follow up rate > 80%), or description provided of those lost	8	Low Risk
Khullar et al., 2023	Somewhat representative of the average patient/community member with Long Covid (eg, age, social class, ethnicity etc.) in the community	Drawn from the same community as the exposed cohort	Secure record (eg medical records)	No	Both of the above	Record linkage (e.g., identified through ICD codes on database records)	Yes (i.e., >4 weeks)	No statement	7	Low Risk
Li et al., 2023	Somewhat representative of the average	Drawn from the same community as	Secure record (eg	No	Both of the above	Self-report (i.e., no reference to original medical	Yes (i.e., >4 weeks)	No statement	7	Low Risk

	patient/community member with Long Covid (eg, age, social class, ethnicity etc.) in the community	the exposed cohort	medical records)			records or laboratory reports to confirm the outcome)				
Marasco et al., 2022	Somewhat representative of the average patient/community member with Long Covid (eg, age, social class, ethnicity etc.) in the community	Drawn from the same community as the exposed cohort	Secure record (eg medical records)	No	Both of the above	Self-report (i.e., no reference to original medical records or laboratory reports to confirm the outcome)	Yes (i.e., >4 weeks)	No statem ent	7	Low Risk
Nehme et al., 2022	Somewhat representative of the average patient/community member with Long Covid (eg, age, social class, ethnicity etc.) in the community	Drawn from the same community as the exposed cohort	Secure record (eg medical records)	No	Both of the above	Self-report (i.e., no reference to original medical records or laboratory reports to confirm the outcome)	Yes (i.e., >4 weeks)	No statem ent	7	Low Risk
Newlan ds et al., 2023	Somewhat representative of the average patient/community member with Long Covid (eg, age, social class, ethnicity etc.) in the community	Drawn from the same community as the exposed cohort	Secure record (eg medical records)	No		Self-report (i.e., no reference to original medical records or laboratory reports to confirm the outcome)	Yes (i.e., >4 weeks)	No statem ent	5	Medium Risk
Noviello et al., 2022	Somewhat representative of the average patient/community member with Long Covid (eg, age, social class, ethnicity etc.) in the community	Drawn from the same community as the exposed cohort	Secure record (eg medical records)	No	Both of the above	Self-report (i.e., no reference to original medical records or laboratory reports to confirm the outcome)	Yes (i.e., >4 weeks)	No statem ent	7	Low Risk
Nugawe la et al., 2022	Truly representative of the average	Drawn from the same community as	Secure record (eg	No	Both of the above	Self-report (i.e., no reference to original medical	Yes (i.e., >4 weeks)	No statem ent	6	Medium Risk

	patient/community member with Long Covid (eg, age, social class, ethnicity etc.) in the community	the exposed cohort	medical records)			records or laboratory reports to confirm the outcome)				
Pagen et al., 2023	Somewhat representative of the average patient/community member with Long Covid (eg, age, social class, ethnicity etc.) in the community	Drawn from the same community as the exposed cohort	Secure record (eg medical records)	No	Both of the above	Self-report (i.e., no reference to original medical records or laboratory reports to confirm the outcome)	Yes (i.e., >4 weeks)	No statem ent	7	Low Risk
Park, et al. 2021	Somewhat representative of the average patient/community member with Long Covid (eg, age, social class, ethnicity etc.) in the community	Drawn from the same community as the exposed cohort	Secure record (eg medical records)	No	Both of the above	Record linkage (e.g., identified through ICD codes on database records)	Yes (i.e., >4 weeks)	No statem ent	7	Low Risk
Pihlaja et al., 2023	Selected groups of patients/communit y members (eg, health care workers, pregnant populations, patients attending specialist Long Covid clinics)	No description of the derivation of the non exposed cohort	Secure record (eg medical records)	No	Both of the above	No description	Yes (i.e., >4 weeks)	No statem ent	4	Medium Risk
PintoPer eira et al., 2023	Somewhat representative of the average patient/community member with Long Covid (eg, age, social class, ethnicity etc.) in the community	Drawn from the same community as the exposed cohort	Secure record (eg medical records)	No	Both of the above	Self-report (i.e., no reference to original medical records or laboratory reports to confirm the outcome)	Yes (i.e., >4 weeks)	Subject s lost to follow up unlikely to introdu ce bias (i.e., follow up rate	8	Low Risk

								> 80%), or description provided of those lost		
Radtke, et al. 2021	Somewhat representative of the average patient/community member with Long Covid (eg, age, social class, ethnicity etc.) in the community	Drawn from the same community as the exposed cohort	Secure record (eg medical records)	No		Self-report (i.e., no reference to original medical records or laboratory reports to confirm the outcome)	Yes (i.e., >4 weeks)	No statement	5	Medium Risk
Rao et al., 2022	Truly representative of the average patient/community member with Long Covid (eg, age, social class, ethnicity etc.) in the community	Drawn from the same community as the exposed cohort	Secure record (eg medical records)	No	Both of the above	Record linkage (e.g., identified through ICD codes on database records)	Yes (i.e., >4 weeks)	No statement	7	Low Risk
Rivera-Izquierdo et al., 2022	Somewhat representative of the average patient/community member with Long Covid (eg, age, social class, ethnicity etc.) in the community	Drawn from the same community as the exposed cohort	Secure record (eg medical records)	no	Both of the above	Self-report (i.e., no reference to original medical records or laboratory reports to confirm the outcome)	Yes (i.e., >4 weeks)	No statement	7	Low Risk
Roge, et al. 2021	Somewhat representative of the average patient/community member with Long Covid (eg, age, social class, ethnicity etc.) in the community	Drawn from a different source	Structured interview	No	Study controls for age and sex in the analysis	Self-report (i.e., no reference to original medical records or laboratory reports to confirm the outcome)	Yes (i.e., >4 weeks)	No statement	4	Medium Risk

Seery et al., 2023	Somewhat representative of the average patient/community member with Long Covid (eg, age, social class, ethnicity etc.) in the community	Drawn from the same community as the exposed cohort	Secure record (eg medical records)	No	Both of the above	Self-report (i.e., no reference to original medical records or laboratory reports to confirm the outcome)	Yes (i.e., >4 weeks)	Subjects lost to follow up unlikely to introduce bias (i.e., follow up rate > 80%), or description provided of those lost	8	Low Risk
Shah et al., 2023	Somewhat representative of the average patient/community member with Long Covid (eg, age, social class, ethnicity etc.) in the community	Drawn from the same community as the exposed cohort	Secure record (eg medical records)	No	Both of the above	Record linkage (e.g., identified through ICD codes on database records)	Yes (i.e., >4 weeks)	Subjects lost to follow up unlikely to introduce bias (i.e., follow up rate > 80%), or description provided of those lost	8	Low Risk
Sneller et al., 2022	Selected groups of patients/community members (eg,	Drawn from the same community as	Structured interview	No	Study controls for age and sex in the analysis	Self-report (i.e., no reference to original medical	Yes (i.e., >4 weeks)	No statement	4	Medium Risk

	health care workers, pregnant populations, patients attending specialist Long Covid clinics)	the exposed cohort				records or laboratory reports to confirm the outcome)				
Subramanian et al., 2022	Truly representative of the average patient/community member with Long Covid (eg, age, social class, ethnicity etc.) in the community	Drawn from the same community as the exposed cohort	Secure record (eg medical records)	No	Both of the above	Record linkage (e.g., identified through ICD codes on database records)	Yes (i.e., >4 weeks)	No statement	7	Low Risk
Thors et al., 2024	Somewhat representative of the average patient/community member with Long Covid (eg, age, social class, ethnicity etc.) in the community	Drawn from the same community as the exposed cohort	Secure record (eg medical records)	No	Study controls for age and sex in the analysis	Self-report (i.e., no reference to original medical records or laboratory reports to confirm the outcome)	Yes (i.e., >4 weeks)	Subjects lost to follow up unlikely to introduce bias (i.e., follow up rate > 80%), or description provided of those lost	7	Low Risk
Tisler et al., 2022	Truly representative of the average patient/community member with Long Covid (eg, age, social class, ethnicity etc.) in the community	Drawn from the same community as the exposed cohort	Secure record (eg medical records)	No	Both of the above	Record linkage (e.g., identified through ICD codes on database records)	Yes (i.e., >4 weeks)	No statement	7	Low Risk

Vander Maaden et al., 2023	Somewhat representative of the average patient/community member with Long Covid (eg, age, social class, ethnicity etc.) in the community	Drawn from the same community as the exposed cohort	Secure record (eg medical records)	No	Both of the above	Self-report (i.e., no reference to original medical records or laboratory reports to confirm the outcome)	Yes (i.e., >4 weeks)	Subjects lost to follow up unlikely to introduce bias (i.e., follow up rate > 80%), or description provided of those lost	8	Low Risk
VanHerck et al., 2024	Somewhat representative of the average patient/community member with Long Covid (eg, age, social class, ethnicity etc.) in the community	Drawn from the same community as the exposed cohort	Secure record (eg medical records)	No	Both of the above	Self-report (i.e., no reference to original medical records or laboratory reports to confirm the outcome)	Yes (i.e., >4 weeks)	Subjects lost to follow up unlikely to introduce bias (i.e., follow up rate > 80%), or description provided of those lost	8	Low Risk
Xiong et al., 2021	Somewhat representative of the average	Drawn from a different source	Secure record (eg	No	0	Self-report (i.e., no reference to original medical	Yes (i.e., >4 weeks)	Subjects lost to	6	Medium Risk

	patient/community member with Long Covid (eg, age, social class, ethnicity etc.) in the community		medical records)			records or laboratory reports to confirm the outcome)		follow up unlikely to introduce bias (i.e., follow up rate > 80%), or description provided of those lost		
Xu et al., 2022	Somewhat representative of the average patient/community member with Long Covid (eg, age, social class, ethnicity etc.) in the community	Drawn from the same community as the exposed cohort	Secure record (eg medical records)	No	Both of the above	Record linkage (e.g., identified through ICD codes on database records)	Yes (i.e., >4 weeks)	Subjects lost to follow up unlikely to introduce bias (i.e., follow up rate > 80%), or description provided of those lost	8	Low Risk
Zhang et al., 2024	Truly representative of the average patient/community member with Long Covid (eg, age,	Drawn from the same community as the exposed cohort	Secure record (eg medical records)	No	Both of the above	Record linkage (e.g., identified through ICD codes on database records)	Yes (i.e., >4 weeks)	No statement	7	Low Risk

	social class, ethnicity etc.) in the community									
Zhao et al., 2024	Somewhat representative of the average patient/community member with Long Covid (eg, age, social class, ethnicity etc.) in the community	Drawn from the same community as the exposed cohort	Secure record (eg medical records)	No	Study controls for age and sex in the analysis	Record linkage (e.g., identified through ICD codes on database records)	Yes (i.e., >4 weeks)	No statem ent	6	Medium Risk

To assess the risk of bias of included studies, an adjusted version of the Newcastle-Ottawa Scale was used. Each study received an overall risk of bias rating of low, medium or high. *Both of the above referes to (i) Study controls for age and sex in the analysis; (ii) Study controls for any additional factors (e.g. co-morbidities, race/ethnicity, socio-economic status, education).

Supplementary Table 5 : Meta-regressions to assess if study level characteristics were associated with estimated effect size

Study level variable	N studies	Coefficient (95% CI)	p-value
**% Male**			
Fatigue	29	0.023 (-0.023, 0.069)	0.314
**Fever**	**10**	**0.075 (0.051, 0.099)**	**<0.001**
Menstrual changes	3	Insufficient observations	
Change in appetite	11	-0.001 (-0.071, 0.068)	0.968
Weight loss	9	0.069 (-0.017, 0.155)	0.124
Dizziness	18	0.003 (-0.009, 0.015)	0.612
Muscle weakness/pain	20	0.002 (-0.020, 0.026)	0.832
Joint pain	13	0.002 (-0.018, 0.023)	0.795
Pain/discomfort	7	0.049 (-0.015, 0.114)	0.110
Breathlessness	23	-0.009 (-0.060, 0.043)	0.730
Indigestion	3	Insufficient observations	
**Cough**	**19**	**0.060 (0.005, 0.114)**	**0.033**
Chest paint/tightness	20	0.007 (-0.031, 0.045)	0.702
Palpitations	10	0.011 (-0.056, 0.077)	0.722
Hoarse voice	5	0.128 (-0.122, 0.378)	0.202
Diarrhoea	10	-0.016 (-0.034, 0.002)	0.077
Constipation	5	-0.007 (-0.049, 0.035)	0.637
Gastro-intestinal problems	8	-0.005 (-0.045, 0.035)	0.761
Vomiting/nausea	16	0.048 (0.015, 0.081)	0.007
Abdominal pain	13	0.001 (-0.038, 0.039)	0.977
Sore throat	12	0.003 (-0.002, 0.110)	0.056
Nasal symptoms	10	0.062 (-0.042, 0.165)	0.209
Headache/migraine	29	0.003 (-0.017, 0.023)	0.755
Loss of smell	17	-0.006 (-0.043, 0.031)	0.736
Loss of taste	12	-0.005 (-0.042, 0.031)	0.749
Affected vision/eye problems	12	0.007 (-0.008, 0.022)	0.336
Affected hearing	10	0.000 (-0.004, 0.004)	0.976
Affected sleep	20	0.011 (-0.054, 0.075)	0.737
Anxiety	16	0.009 (-0.005, 0.022)	0.186
Depression	16	0.009 (-0.004, 0.022)	0.148
PTSD	4	0.014 (-0.235, 0.263)	0.832
Impaired mobility/walking	4	-0.029 (-0.079, 0.022)	0.135
Vertigo/balance issues	5	-0.004 (-0.084, 0.077)	0.892
Paraethesia	8	0.001 (-0.009, 0.012)	0.769
Chills/ shivers	3	Insufficient observations	
Impaired memory	11	-0.007 (-0.041, 0.027)	0.654
Poor concentration	10	0.006 (-0.114, 0.126)	0.913
Cognitive dysfunction/impairment	8	0.002 (-0.068, 0.073)	0.927
Confusion/brain fog	8	-0.016 (-0.112, 0.080)	0.698
At least 1 symptom at follow-up	11	-0.009 (-0.089, 0.070)	0.799
**Hair loss/alopecia**	**10**	**-0.070 (-0.130, -0.011)**	**0.026**
Dermatological problems	16	0.004 (-0.064, 0.072)	0.896
**Mean follow-up (days)**			
Fatigue	24	-0.001 (-0.004, 0.001)	0.279
Fever	11	-0.001 (-0.005, 0.003)	0.494
Menstrual changes	3	Insufficient observations	
Change in appetite	9	0.001 (-0.001 , 0.003)	0.383
Weight loss	3	Insufficient observations	
Dizziness	16	0.001 (-0.000, 0.002)	0.228
Muscle weakness/pain	18	0.002 (-0.001, 0.004)	0.142
Joint pain	12	0.001 (-0.001, 0.002)	0.219
Pain/discomfort	6	0.002 (-0.000, 0.004)	0.052
Breathlessness	18	-0.001 (-0.003, 0.001)	0.360
Indigestion	3	Insufficient observations	
Cough	17	0.001 (-0.002, 0.003)	0.515
Chest paint/tightness	18	0.001 (-0.000, 0.003)	0.054
Palpitations	9	0.002 (0.001, 0.002)	0.009
Hoarse voice	4	-0.005 (-0.030, 0.019)	0.484

Diarrhoea	10	0.000 (-0.002, 0.003)	0.718
Constipation	4	0.000 (-0.001, 0.001)	0.953
Gastro-intestinal problems	7	0.002 (-0.001, 0.004)	0.118
Vomiting/nausea	14	0.001 (-0.001, 0.003)	0.349
Abdominal pain	10	-0.000 (-0.001, 0.001)	0.856
Sore throat	12	0.003 (-0.000, 0.006)	0.072
Nasal symptoms	8	-0.002 (-0.008, 0.004)	0.442
Headache/migraine	24	0.001 (-0.001, 0.003)	0.170
Loss of smell	14	0.002 (-0.001, 0.006)	0.182
Loss of taste	10	0.000 (-0.004, 0.004)	0.858
Affected vision/eye problems	12	0.001 (-0.001, 0.003)	0.211
Affected hearing	10	0.000 (-0.001, 0.001)	0.177
Affected sleep	17	0.000 (-0.003, 0.003)	0.970
Anxiety	14	0.001 (-0.000, 0.003)	0.121
Depression	15	0.001 (0.001, 0.003)	0.278
PTSD	3	Insufficient observations	
Impaired mobility/walking	4	-0.001 (-0.003, 0.001)	0.143
Vertigo/balance issues	4	0.001 (-0.002, 0.003)	0.231
Paraethesia	7	0.001 (-0.000, 0.002)	0.116
Chills/ shivers	4	-0.005 (-0.012, 0.002)	0.086
Impaired memory	9	-0.001 (-0.006, 0.004)	0.623
Poor concentration	7	0.002 (-0.004, 0.008)	0.434
Cognitive dysfunction/impairment	3	Insufficient observations	
Confusion/brain fog	9	-0.001 (-0.004, 0.003)	0.696
At least 1 symptom at follow-up	9	-0.001 (-0.005, 0.004)	0.759
Hair loss/alopecia	10	-0.000 (-0.004, 0.004)	0.840
Dermatological problems	14	0.003 (-0.000, 0.007)	0.050
Mean age (years)			
Fatigue	21	-0.012 (-0.03, 0.009)	0.240
Fever	7	-0.009 (-0.038, 0.021)	0.496
Menstrual changes	3	Insufficient observations	
Change in appetite	9	-0.001 (-0.043, 0.030)	0.691
Weight loss	4	-0.032 (-0.074, 0.011)	0.084
Dizziness	14	-0.003 (0.024, 0.019)	0.799
Muscle weakness/pain	18	-0.000 (-0.023, 0.022)	0.975
Joint pain	13	-0.015 (-0.057, 0.027)	0.445
Pain/discomfort	6	0.008 (-0.031, 0.047)	0.592
Breathlessness	16	-0.007 (-0.034, 0.021)	0.598
Indigestion	3	Insufficient observations	
Cough	16	-0.004 (-0.032, 0.025)	0.775
Chest paint/tightness	16	0.005 (0.016, 0.026)	0.631
Palpitations	9	0.004 (-0.031, 0.040)	0.778
Hoarse voice	4	0.011 (-0.188, 0.210)	0.836
Diarrhoea	10	-0.10 (-0.034, 0.014)	0.366
Constipation	5	-0.017 (-0.051, 0.017)	0.214
Gastro-intestinal problems	6	0.010 (-0.027, 0.048)	0.487
Vomiting/nausea	15	0.008 (-0.014, 0.029)	0.455
Abdominal pain	10	-0.027 (-0.050, -0.003)	0.028
Sore throat	9	0.018 (-0.029, 0.065)	0.392
Nasal symptoms	9	-0.020 (-0.055, 0.015)	0.223
Headache/migraine	21	-0.017 (-0.039, 0.005)	0.121
Loss of smell	14	0.007 (-0.026, 0.039)	0.652
Loss of taste	12	-0.028 (-0.077, 0.021)	0.229
Affected vision/eye problems	9	0.003 (-0.030, 0.035)	0.846
Affected hearing	9	0.004 (0.000, 0.008)	0.045
Affected sleep	15	0.008 (-0.029, 0.044)	0.660
Anxiety	12	0.007 (-0.005, 0.019)	0.249
Depression	13	0.014 (-0.003, 0.030)	0.102
PTSD	3	Insufficient observations	
Impaired mobility/walking	3	Insufficient observations	
Vertigo/balance issues	5	-0.005 (-0.065, 0.055)	0.819
Paraethesia	8	-0.005 (-0.036, 0.026)	0.728
Chills/ shivers	3	Insufficient observations	
Impaired memory	11	-0.006 (-0.043, 0.029)	0.700

Poor concentration	9	0.008 (-0.042, 0.057)	0.725
Cognitive dysfunction/impairment	4	0.050 (-0.081, 0.181)	0.245
Confusion/brain fog	4	-0.018 (-0.251, 0.214)	0.770
At least 1 symptom at follow-up	11	0.007 (-0.025, 0.039)	0.630
Hair loss/alopecia	10	0.018 (-0.028, 0.063)	0.397
Dermatological problems	16	-0.010 (-0.045, 0.025)	0.529

Post-hoc meta-regression analysis (2-sided and no adjustment made for multiple comparisons).

Supplementary Fig. 1: Forest plot of pooled relative risks for each symptom (studies of hospitalised participants at time of covid infection)

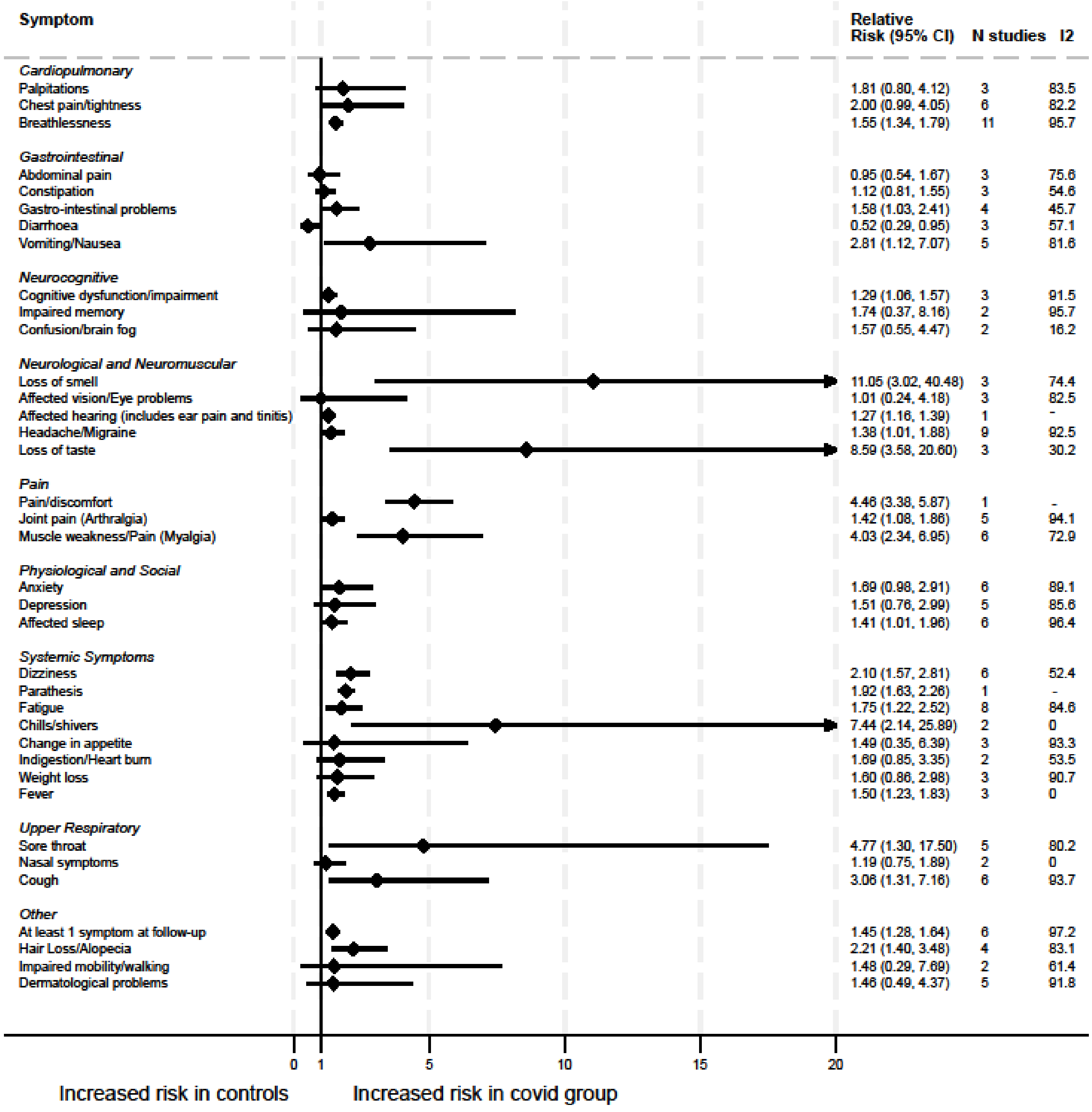

Illustrates pooled relative risks and associated 95% confidence intervals for each symptom using random effects models. All tests were 2-sided and no adjustment was made for multiple comparisons. Heterogeneity was assessed using Higgins I^2 statistic (I^2 between 75% - 100% indicates considerable between study heterogeneity).

Supplementary Fig. 2: Forest plot of pooled relative risks for each symptom (studies of non-hospitalised participants at time of covid infection)

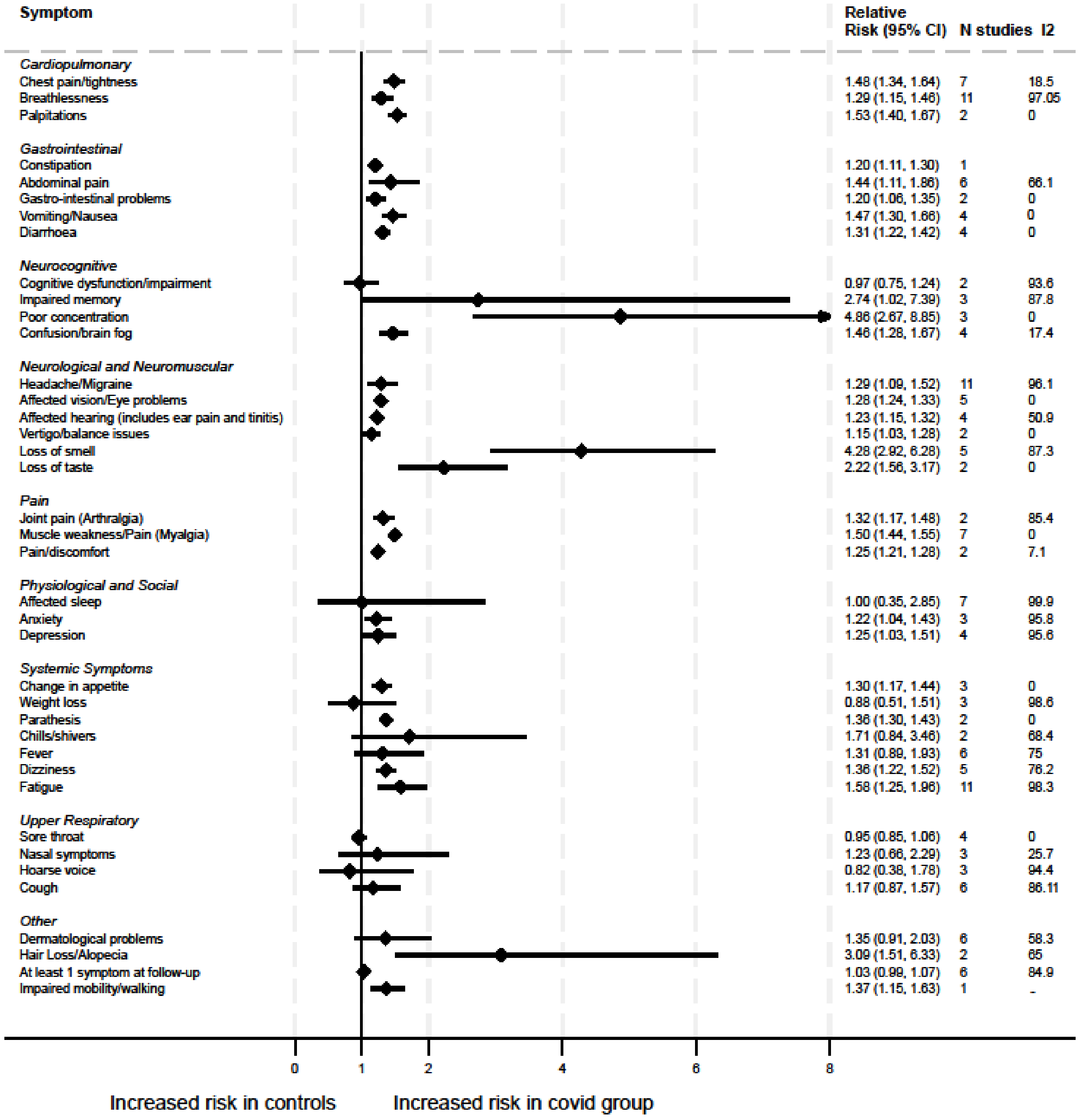

Illustrates pooled relative risks and associated 95% confidence intervals for each symptom using random effects models. All tests were 2-sided and no adjustment was made for multiple comparisons. Heterogeneity was assessed using Higgins I^2 statistic (I^2 between 75% - 100% indicates considerable between study heterogeneity).

Supplementary Fig. 3: Forest plot of pooled relative risks for each symptom (studies of hospitalised and non-hospitalised participants at time of covid infection)

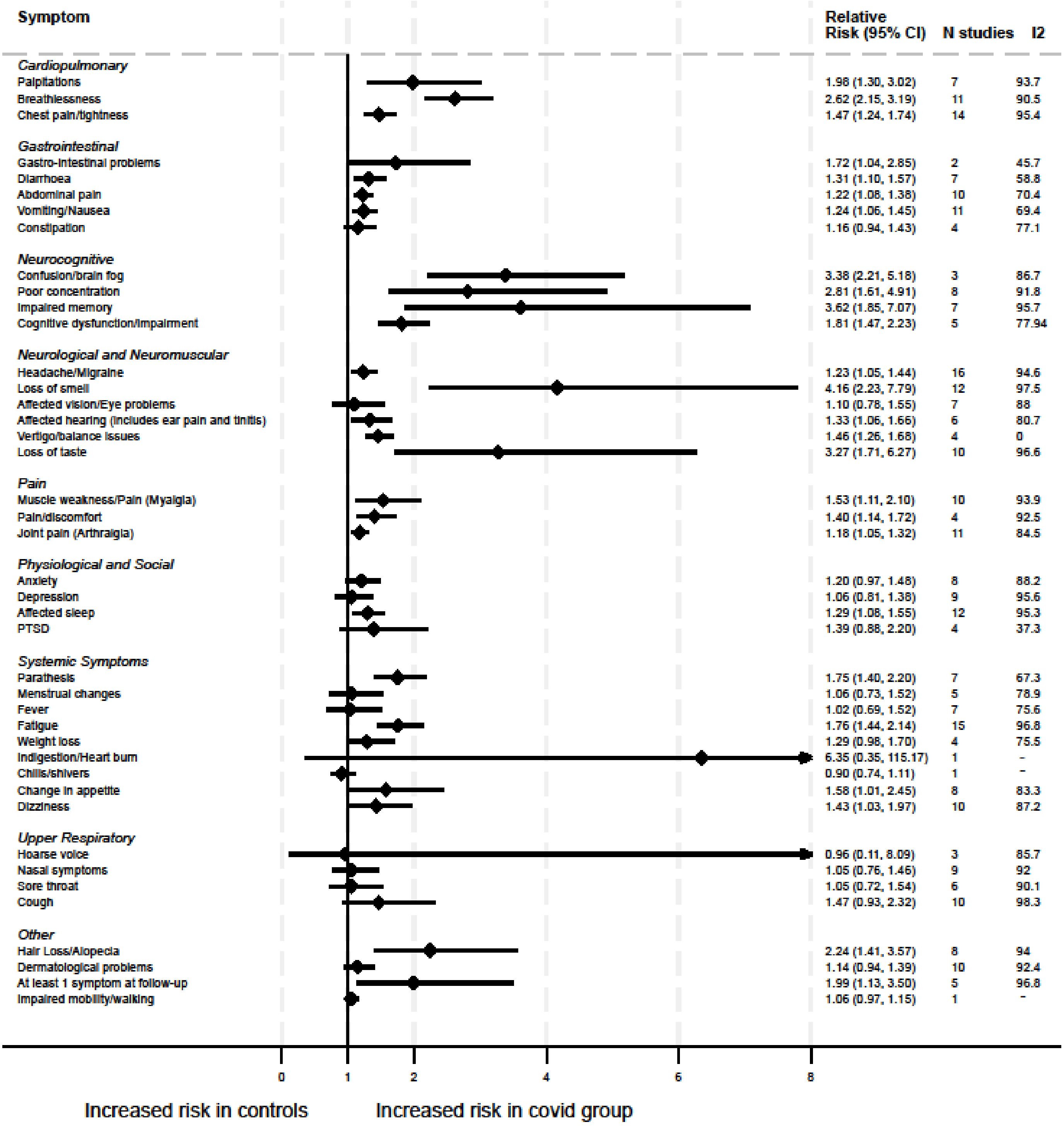

Illustrates pooled relative risks and associated 95% confidence intervals for each symptom using random effects models. All tests were 2-sided and no adjustment was made for multiple comparisons. Heterogeneity was assessed using Higgins I² statistic (I^2 between 75% - 100% indicates considerable between study heterogeneity).

References for Included Studies

1. Adler, L., *et al.* Long-COVID in patients with a history of mild or asymptomatic SARS-CoV-2 infection: a Nationwide Cohort Study. *Scandinavian Journal of Primary Health Care* **40**, 342-349 (2022).
2. Ahn, B., Choi, S.H. & Yun, K.W. Non-neuropsychiatric Long COVID Symptoms in Children Visiting a Pediatric Infectious Disease Clinic After an Omicron Surge. *Pediatr Infect Dis J* **42**, e143-e145 (2023).
3. Albtoosh, A.S., *et al.* New symptoms and prevalence of postacute COVID-19 syndrome among nonhospitalized COVID-19 survivors. *Scientific reports* **12**, 16921 (2022).
4. Ali, M., Bonna, A.S., Sarkar, A.S. & Islam, A. Is Coronavirus Infection Associated With Musculoskeletal Health Complaints? Results From a Comprehensive Case-Control Study. *J Prim Care Community Health* **13**, 21501319221114259 (2022).
5. Askarian, M., *et al.* Epidemiological and clinical characteristics of long COVID-19 among Iranians: A community-based study in southern Iran. *BMC Public Health* **24**, 2007 (2024).
6. Barboza-Solis, C., *et al.* COVID-19 and long-term impact on symptoms and Health-Related Quality of Life in Costa Rica: the RESPIRA cohort study. *BMC Infectious Diseases* **24**, 557 (2024).
7. Bergia, M., *et al.* Comparative study shows that 1 in 7 Spanish children with COVID-19 symptoms were still experiencing issues after 12 weeks. *Acta paediatrica (Oslo, Norway : 1992)* **111**, 1573-1582 (2022).
8. Binswanger, I.A., *et al.* Assessing the association between antibody status and symptoms of long COVID: A multisite study. *PLoS One* **19**, e0304262 (2024).
9. Blomberg, B., *et al.* Long COVID in a prospective cohort of home-isolated patients. *Nature Medicine* **27**, 1607-1613 (2021).
10. Boscolo-Rizzo, P., *et al.* High prevalence of long-term olfactory, gustatory, and chemesthesis dysfunction in post-COVID-19 patients: a matched case-control study with one-year follow-up using a comprehensive psychophysical evaluation. *Rhinology* **59**, 517-527 (2021).
11. Cai, J., *et al.* A one-year follow-up study of systematic impact of long COVID symptoms among patients post SARS-CoV-2 omicron variants infection in Shanghai, China. *Emerg Microbes Infect* **12**, 2220578 (2023).
12. de Bruijn, S., *et al.* Lower prevalence of post-Covid-19 Condition following Omicron SARS-CoV-2 infection. *Heliyon* **10**, e28941 (2024).
13. Desgranges, F., *et al.* Post-COVID-19 Syndrome in Outpatients: a Cohort Study. *Journal of general internal medicine* **37**, 1943-1952 (2022).
14. Fjelltveit, E.B., *et al.* Symptom Burden and Immune Dynamics 6 to 18 Months Following Mild Severe Acute Respiratory Syndrome Coronavirus 2 Infection (SARS-CoV-2): A Case-control Study. *Clinical Infectious Diseases* **76**, e60-e70 (2023).
15. Funk, A.L., *et al.* Post–COVID-19 Conditions Among Children 90 Days After SARS-CoV-2 Infection. *JAMA Network Open* **5**, e2223253-e2223253 (2022).
16. Golla, R., *et al.* Long-term Gastrointestinal Sequelae Following COVID-19: A Prospective Follow-up Cohort Study. *Clinical gastroenterology and hepatology : the official clinical practice journal of the American Gastroenterological Association* **21**, 789-796.e781 (2023).
17. Hastie, C.E., *et al.* True prevalence of long-COVID in a nationwide, population cohort study. *Nat Commun* **14**, 7892 (2023).
18. Hernandez-Romieu, A.C., *et al.* Prevalence of Select New Symptoms and Conditions Among Persons Aged Younger Than 20 Years and 20 Years or Older at 31 to 150 Days After Testing Positive or Negative for SARS-CoV-2. *JAMA Network Open* **5**, e2147053-e2147053 (2022).
19. Horberg, M.A., *et al.* Post-acute sequelae of SARS-CoV-2 with clinical condition definitions and comparison in a matched cohort. *Nature communications* **13**, 5822 (2022).

20. Hosozawa, M., *et al.* Prevalence and risk factors of post-coronavirus disease 2019 condition among children and adolescents in Japan: A matched case-control study in the general population. *Int J Infect Dis* **143**, 107008 (2024).
21. Huang, L., *et al.* Health outcomes in people 2 years after surviving hospitalisation with COVID-19: a longitudinal cohort study. *The Lancet Respiratory Medicine* **10**, 863-876 (2022).
22. Huynh, D.H.T., *et al.* Clinical Characteristics and Outcomes of Long COVID-19 Hospitalized Children in Vietnam. *Pediatr Infect Dis J* **42**, e366-e368 (2023).
23. Iba, A., *et al.* Prevalence of and Risk Factors for Post-COVID-19 Condition during Omicron BA.5-Dominant Wave, Japan. *Emerg Infect Dis* **30**, 1380-1389 (2024).
24. Khullar, D., *et al.* Racial/Ethnic Disparities in Post-acute Sequelae of SARS-CoV-2 Infection in New York: an EHR-Based Cohort Study from the RECOVER Program. *J Gen Intern Med* **38**, 1127-1136 (2023).
25. Li, J., Nadua, K., Chong, C.Y. & Yung, C.F. Long COVID prevalence, risk factors and impact of vaccination in the paediatric population: A survey study in Singapore. *Ann Acad Med Singap* **52**, 522-532 (2023).
26. Marasco, G., *et al.* Prevalence of Gastrointestinal Symptoms in Severe Acute Respiratory Syndrome Coronavirus 2 Infection: results of the Prospective Controlled Multinational GI-COVID-19 Study. *American journal of gastroenterology* **117**, 147-157 (2022).
27. Nehme, M., *et al.* One-year persistent symptoms and functional impairment in SARS-CoV-2 positive and negative individuals. *Journal of internal medicine* **292**, 103-115 (2022).
28. Newlands, F., *et al.* A Cross-Sectional Study of the Health of Emerging Young Adults in England Following a COVID-19 Infection. *J Adolesc Health* **73**, 20-28 (2023).
29. Noviello, D., *et al.* Functional gastrointestinal and somatoform symptoms five months after SARS-CoV-2 infection: A controlled cohort study. *Neurogastroenterology and motility : the official journal of the European Gastrointestinal Motility Society* **34**, e14187 (2022).
30. Nugawela, M.D., *et al.* Predictive model for long COVID in children 3 months after a SARS-CoV-2 PCR test. *BMC medicine* **20**, 465 (2022).
31. Pagen, D.M.E., *et al.* Prevalence of Long-term Symptoms Varies When Using Different Post-COVID-19 Definitions in Positively and Negatively Tested Adults: The PRIME Post-COVID Study. *Open Forum Infect Dis* **10**, ofad471 (2023).
32. Park, H.Y., Song, I.A. & Oh, T.K. Insomnia Disorder Among Coronavirus Disease Survivors: A South Korean Nationwide Cohort Study. *Psychiatry Investig* **18**, 1082-1090 (2021).
33. Pihlaja, R.E., *et al.* Associations of subjective and objective cognitive functioning after COVID-19: A six-month follow-up of ICU, ward, and home-isolated patients. *Brain, Behavior, and Immunity - Health* **27**(2023).
34. Pinto Pereira, S.M., *et al.* Symptom Profiles of Children and Young People 12 Months after SARS-CoV-2 Testing: A National Matched Cohort Study (The CLoCk Study). *Children (Basel)* **10**(2023).
35. Radtke, T., Ulyte, A., Puhan, M.A. & Kriemler, S. Long-term Symptoms After SARS-CoV-2 Infection in Children and Adolescents. *JAMA* **326**, 869-871 (2021).
36. Rao, S., *et al.* Clinical Features and Burden of Postacute Sequelae of SARS-CoV-2 Infection in Children and Adolescents. *JAMA Pediatrics* **176**, 1000-1009 (2022).
37. Rivera-Izquierdo, M., *et al.* Long COVID 12 months after discharge: persistent symptoms in patients hospitalised due to COVID-19 and patients hospitalised due to other causes-a multicentre cohort study. *BMC medicine* **20**, 92 (2022).
38. Roge, I., *et al.* Comparison of Persistent Symptoms After COVID-19 and Other Non-SARS-CoV-2 Infections in Children. *Front Pediatr* **9**, 752385 (2021).

39. Seery, V., *et al.* Persistent symptoms after COVID-19 in children and adolescents from Argentina. *Int J Infect Dis* **129**, 49-56 (2023).
40. Shah, A.D., *et al.* Long Covid symptoms and diagnosis in primary care: A cohort study using structured and unstructured data in The Health Improvement Network primary care database. *PLoS One* **18**, e0290583 (2023).
41. Sneller, M.C., *et al.* A Longitudinal Study of COVID-19 Sequelae and Immunity: Baseline Findings. *Annals of Internal Medicine* **175**, 969-979 (2022).
42. Subramanian, A., *et al.* Symptoms and risk factors for long COVID in non-hospitalized adults. *Nature medicine* **28**, 1706-1714 (2022).
43. Thors, V., Bjornsdottir, K.L., Love, T.J. & Haraldsson, A. Long COVID in Icelandic Children: A Matched Cohort Study of Nonspecific Symptoms Following SARS-CoV-2 Infection. *Pediatr Infect Dis J* **43**, 226-233 (2024).
44. Tisler, A., *et al.* Post-acute sequelae of COVID-19 among hospitalized patients in Estonia: Nationwide matched cohort study. *PloS one* **17**, e0278057 (2022).
45. van der Maaden, T., *et al.* Prevalence and Severity of Symptoms 3 Months After Infection With SARS-CoV-2 Compared to Test-Negative and Population Controls in the Netherlands. *J Infect Dis* **227**, 1059-1067 (2023).
46. Van Herck, M., *et al.* Impact of post-COVID-19 condition on health status and activities of daily living: the PRIME post-COVID study. *Thorax* **79**, 457-464 (2024).
47. Xiong, Q., *et al.* Clinical sequelae of COVID-19 survivors in Wuhan, China: a single-centre longitudinal study. *Clin Microbiol Infect* **27**, 89-95 (2021).
48. Xu, E., Xie, Y. & Al-Aly, Z. Long-term neurologic outcomes of COVID-19. *Nature medicine* **28**, 2406-2415 (2022).
49. Zhang, Y., *et al.* Association between SARS-CoV-2 infection and select symptoms and conditions 31 to 150 days after testing among children and adults. *BMC Infectious Diseases* **24**, 181 (2024).
50. Zhao, S., *et al.* Long COVID is associated with severe cognitive slowing: a multicentre cross-sectional study. *eClinicalMedicine* **68**(2024).
